# Multiple stressors produce differential transcriptomic patterns in a stream-dwelling salamander

**DOI:** 10.1186/s12864-019-5814-y

**Published:** 2019-06-11

**Authors:** Timothy A. Clay, Michael A. Steffen, Michael L. Treglia, Carolyn D. Torres, Ana Lilia Trujano-Alvarez, Ronald M. Bonett

**Affiliations:** 1 Department of Biological Science, University of Tulsa, Tulsa, OK 74104 USA; 2 Present Address: Department of Biological Sciences, Nicholls State University, Thibodaux, LA 70310 USA; 3 Present Address: Department of Biological Sciences, University of New Hampshire, Durham, NH 03824 USA; 4 Present Address: The Nature Conservancy, New York, NY 10001 USA

**Keywords:** Biomarkers, Transcriptome, Stress, Salamander

## Abstract

**Background:**

Global biodiversity is decreasing at an alarming rate and amphibians are at the forefront of this crisis. Understanding the factors that negatively impact amphibian populations and effectively monitoring their health are fundamental to addressing this epidemic. Plasma glucocorticoids are often used to assess stress in amphibians and other vertebrates, but these hormones can be extremely dynamic and impractical to quantify in small organisms. Transcriptomic responses to stress hormones in amphibians have been largely limited to laboratory models, and there have been few studies on vertebrates that have evaluated the impact of multiple stressors on patterns of gene expression. Here we examined the gene expression patterns in tail tissues of stream-dwelling salamanders (*Eurycea tynerensis*) chronically exposed to the stress hormone corticosterone under different temperature regimes.

**Results:**

We found unique transcriptional signatures for chronic corticosterone exposure that were independent of temperature variation. Several of the corticosterone responsive genes are known to be involved in immune system response (*LY-6E*), oxidative stress (*GSTM2* and *TRX*), and tissue repair (*A2M* and *FX*)*.* We also found many genes to be influenced by temperature (*CIRBP, HSC71, HSP40, HSP90, HSP70, ZNF593*). Furthermore, the expression patterns of some genes (*GSTM2, LY-6E, UMOD, ZNF593, CIRBP, HSP90*) show interactive effects of temperature and corticosterone exposure, compared to each treatment alone. Through a series of experiments we also showed that stressor induced patterns of expression were largely consistent across ages, life cycle modes, and tissue regeneration.

**Conclusions:**

Outside of thermal stressors, the application of transcriptomes to monitor the health of non-human vertebrate systems has been vastly underinvestigated. Our study suggests that transcriptomic patterns harbor stressor specific signatures that can be highly informative for monitoring the diverse stressors of amphibian populations.

**Electronic supplementary material:**

The online version of this article (10.1186/s12864-019-5814-y) contains supplementary material, which is available to authorized users.

## Background

Environmental stressors such as habitat degradation, climate change, disease, and invasive species are central to the loss of global biodiversity [1, 2]. Effective conservation and management requires understanding when species cannot cope with environmental conditions [3]. Organismal systems potentially express many signs of enduring stress, but the set of metrics commonly analyzed to assess the health of wildlife are typically limited [4].

In vertebrates, “stress” can activate the hypothalamic-pituitary-interrenal (or adrenal) axis (HPI-axis), leading to the production of elevated plasma glucocorticoids, and these hormones have been widely used for stress assessment [5–8]. However, glucocorticoid levels can be difficult or impossible to measure in some organisms [9, 10] and may not be indicative of underlying chronic (long-term) stress [11–13]. Genes that are regulated directly or indirectly by “stress hormones” or stressors themselves may offer a potentially rich source of informative biomarkers for monitoring population health and understanding adaptation. The transcriptomic responses of climatic variables, specifically temperature, have been well-studied for more than a decade [14, 15], but wildlife are subject to a wide range of stressors [1, 2], some of which may be exacerbated by changing climate [16–18]. Nevertheless, only a few studies have examined the transcriptional responses of animals to multiple stressors [18–21].

Amphibians are at the forefront of the biodiversity crisis and are likely threatened by multiple stressors (habitat loss, disease, climate change) [2, 22–25]. Many amphibian species appear to be declining in relatively healthy and undisturbed ecosystems, and the causative agents are often unknown [22, 25]. Most amphibians are small, and whole animals or pools of animals may need to be sacrificed in order to extract sufficient quantities of glucocorticoids for analysis via radioimmunoassay (e.g. [26]). Water-borne [27, 28], urine [29], fecal [30], and keratinized tissue [31] assays have recently been developed as non-invasive means to monitor glucocorticoid levels. However, daily [32], seasonal [33, 34], and life history variation [8, 35] can present additional challenges to understanding how point measurements of glucocorticoid levels relate to stress response. Gene expression responses in amphibians to “stress” hormones such as glucocorticoids have been studied extensively, but primarily focused on understanding how glucocorticoids regulate cellular, developmental, or physiological mechanisms [6, 36–39]. Furthermore, such studies have been based on model organisms (e.g. *Xenopus*). Only a few studies have evaluated the transcriptional responses of amphibians to climatic variables such as temperature [10, 18, 40].

Here we performed transcriptional analyses on adult stream-dwelling salamanders (*Eurycea tynerensis*) exposed to chronic corticosterone (a primary glucocorticoid) and different temperatures regimes. We tested whether transcriptional patterns provide genetic biomarkers for monitoring stress by simulating chronically elevated corticosterone and fluctuating temperature regimes. We further evaluated the robustness of several “stress response” genes across different life stages (adult and larvae), life histories (biphasic and paedomorphic), and to recent tissue regeneration (Table 1). Many of the differentially regulated genes were specific to temperature variation or corticosterone exposure and are known to be associated with cellular and physiological health in humans and biomedical models. Our analyses suggest that transcriptomic approaches may be key to understanding the diverse types of stressors that can impact amphibians.

**Table 1:** List of five gene expression experiments included in this study. This list includes the purpose, material, and conditions of the experiment, variables for each experimental group, and samples sizes (N) for qPCR of candidate genes and RNA-Seq

Experiments (materials and/or conditions)	Group Variables	qPCR N	RNA-Seq N
Test for differential expression by Temp and Cort exposure over 30 days. (all wild-caught, adult, Paedo)	Control, 11 °C (low)	8	6
	100 nM Cort, 11 °C (low)	7	6
	Control, 21 °C (high)	7	6
	100 nM Cort, 21 °C (high)	8	6
Test for differential expression by Cort exposure over 28 days among larvae of alternative life cycle strategies. (lab raised 21 °C, larvae, Paedo & Biphasic)	Control, Paedo (P)	6	
	100 nM Cort, Paedo (P)	6	
	Control, Biphasic (B)	6	
	100 nM Cort, Biphasic (B)	6	
Test for differential expression by Cort exposure over time and after regeneration. (lab raised 21 °C, larvae, Biphasic)	Control, 0 day	6	
	100 nM Cort, 14 day	6	
	100 nM Cort, 28 day	6	
	100 nM Cort, Regen 14 day	6	
Test for differential expression by Cort exposure of tails in culture. (lab raised 21 °C, 48 h, larvae, Paedo)	Control	6	
	100 nM Cort	6	
Test for differential expression in stable (21 °C) vs fluctuating (14 to 28 °C) temps with the same mean (21 °C). (all wild-caught, adults, Paedo & Biphasic)	Stable temp, Paedo (P)	5	
	Fluctuating temp, Paedo (P)	5	
	Stable temp, Biphasic (B)	6	
	Fluctuating temp, Biphasic (B)	6	

## Results

### Transcriptional responses to corticosterone exposure and temperature

**Fig. 1:**
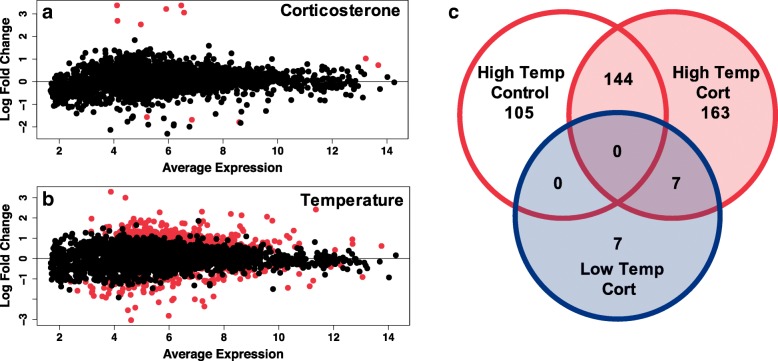
Average log counts per million reads and log fold change of differences in expression for 4082 genes between adult salamanders (*N* = 24) exposed to corticosterone (Cort) vs. control (**a**) and low (11 °C) or high (21 °C) temperatures (**b**). Significant differentially expressed genes are denoted with red circles. Differential gene expression based on pairwise comparisons amongst treatment groups compared to individuals exposed to low temperature control conditions (**c**). Blue circle represents low temperature and red circles represent high temperature treatments. Low temperature control is used as the reference. Corticosterone treatments are shaded

**Fig. 2:**
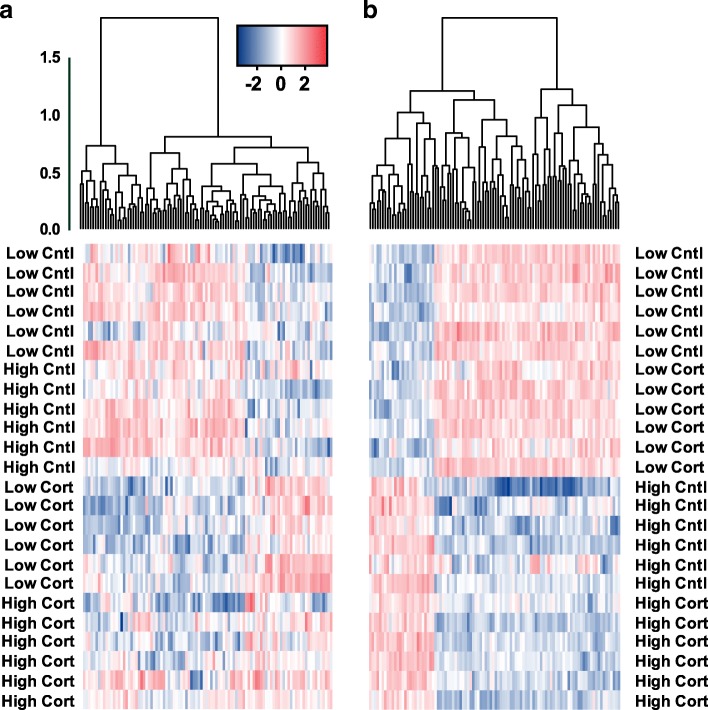
Heatmaps of the top 100 differentially expressed genes in response to (**a**) corticosterone and (**b**) temperature. Hierarchical clustering is based on distance correlation between gene expression values. Expression values are represented by log fold change normalized by library size. Treatment groups are abbreviated as follows: Low Cntl = low temperature control; High Cntl = high temperature control; Low Cort = low temperature corticosterone; High Cort = high temperature corticosterone

Our reference transcriptome for *Eurycea tynerensis* included 4348 identified transcripts (median transcript length: 2207 bp; range: 214 to 17,654 bp; total length: 10,944,985 bp; N50: 3109 bp). Thirty-seven percent of 18.1 million 150 bp paired end RNA-Seq reads mapped to the reference and 4082 transcripts had sufficient coverage for analysis (see Methods section). Our RNA-Seq analyses revealed significant transcriptional responses in tail tissue of aquatic adult salamanders chronically exposed (30 days) to corticosterone at high and low temperatures. Out of 4082 genes, 11 were differentially expressed in response to corticosterone (Fig. 1a), which includes 8 upregulated and 3 downregulated genes (Additional file 2: Table S2). Substantially more genes (837) were differentially expressed by long-term exposure to high versus low temperatures (Fig. 1b). Exposure to high temperature (21 °C) upregulated 665 genes and downregulated 172 genes compared to maintenance at a low temperature (11 °C; Additional file 2: Table S2). Pairwise comparisons between low temperature control treatment and the other three treatments revealed that numerous genes share differential patterns of expression among stress conditions, however many are stressor specific (Fig. 1c; Additional file 2: Table S2). At low temperature, 14 genes were differentially regulated by corticosterone, whereas high temperature plus corticosterone differentially regulated 314 genes. High temperature without corticosterone differentially regulated 249 genes compared to low temperature control (Fig. 1c). Heat map of the 100 most differentially expressed genes shows discrete clustering based on treatments (Fig. 2).

**Fig. 3:**
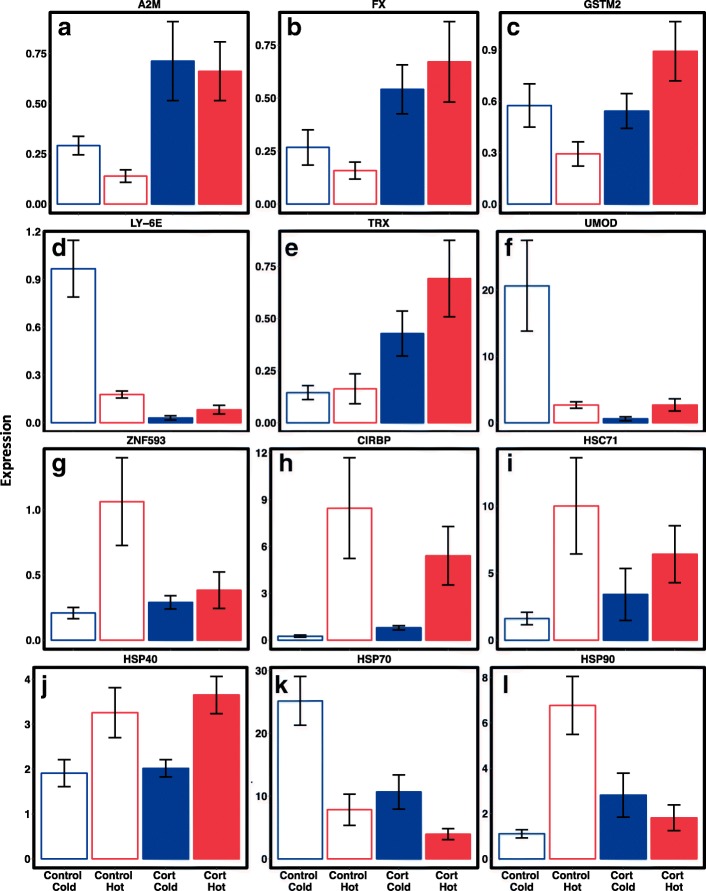
Relative qPCR expression values of 12 genes (corticosterone regulated, **a-f**; and temperature regulated, **g-l**) for adult salamanders (*N* = 30) exposed to low (11 °C) or high (21 °C) temperatures and filtered water (Control) or corticosterone (Cort) for 4 weeks. Blue bars represent low temperature and red bars represent high temperature treatments. Corticosterone treatments are shaded

Quantitative PCR analyses of six corticosterone and six temperature-regulated genes generally reflected our RNA-Seq analysis (Fig. 3). *A2M, FX, GSTM2,* and *TRX* were upregulated, and *LY-6E* and *UMOD* were downregulated in response to corticosterone. Temperature specific genes that were upregulated include *ZNF593*, *CIRBP*, *HSC71*, *HSP40*, and *HSP90*, whereas *HSP70* was downregulated. The effects of corticosterone exposure on some genes (*GSTM2, LY-6E, UMOD, ZNF593, CIRBP, HSP90*) were temperature dependent (Fig. 3).

### Transcriptional responses to corticosterone across life stages and life cycle modes

**Fig. 4:**
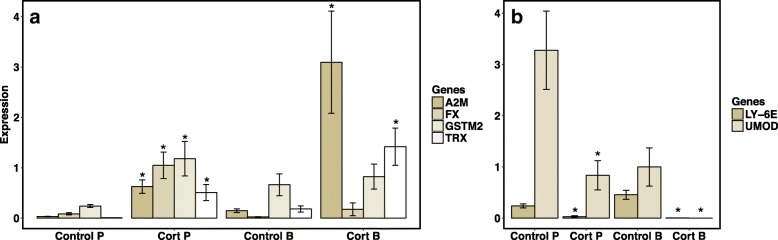
Relative qPCR expression values of four upregulated (**a**) and two downregulated genes (**b**) for paedomorphic (P; *N* = 12) and biphasic (B; *N* = 12) larval salamanders exposed to filtered water (Control) or corticosterone (Cort) for 4 weeks. Significant differences (*P* < 0.05) between corticosterone and control gene expression are denoted with (*)

We found that larvae from both paedomorphic and biphasic populations exposed to corticosterone for 28 days had similar patterns of gene expression to one another (Fig. 4) and to paedomorphic adults (Fig. 3). *LY-6E* and *UMOD* were downregulated, and *A2M* and *TRX* were upregulated with corticosterone exposure (Fig. 4). However, corticosterone had a more dramatic impact on downregulating *LY-6E* and *UMOD* in larvae from biphasic populations compared to larvae from paedomorphic populations (Fig. 4). There was also significant upregulation of *FX* and *GSTM2* in larvae from paedomorphic populations treated with corticosterone, unseen in larvae from biphasic populations. Despite some genes with life cycle dependent responses to chronic corticosterone exposure, other genes (*LY-6E*, *UMOD*, *A2M*, and *TRX*) had consistent patterns of expression across life cycle modes and life stages.

### Transcriptional responses to corticosterone over time and during tissue regeneration

**Fig. 5:**
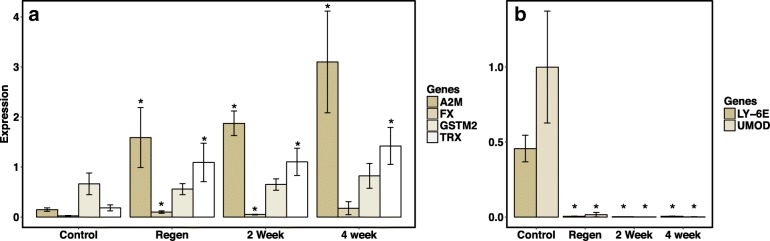
Relative qPCR expression values of four upregulated (**a**) and two downregulated genes (**b**) for larval biphasic salamanders exposed to filtered water (Control; *N* = 6) or corticosterone for 2 weeks (*N* = 6) or 4 weeks (*N* = 6). Salamanders with regenerated tails (Regen; *N* = 6) were exposed to corticosterone for 4 weeks. Significant differences (*P* < 0.05) between corticosterone and control gene expression are denoted with (*)

**Fig. 6:**
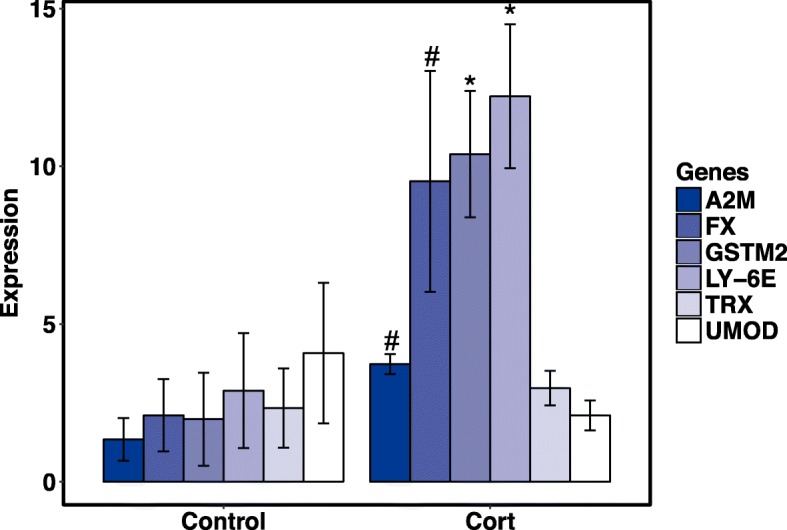
Relative qPCR expression values of six corticosterone sensitive genes from excised tails (*N* = 12) cultured for 48 h at 21 °C. Significant differences (*P* < 0.05) between corticosterone and control gene expression are denoted with (*). Differences less than *P* < 0.01 are denoted with (#)

Circulating levels of glucocorticoids can be dynamic [32, 33, 41], and therefore measurements may be highly variable depending on very recent physiological conditions. In order to assess chronic stress, it may be important to identify markers that more consistently reflect signatures of physiological challenges. We found that the corticosterone induced upregulation of *A2M* and *TRX* and downregulation of *LY-6E* and *UMOD* were constitutively maintained over at least a two-week period (2 weeks to 4 weeks of exposure; Fig. 5). Corticosterone treatment of cultured tails for 48 h demonstrates a similar pattern of upregulation in *GSTM2, A2M* and *FX* (Fig. 6), which were also upregulated by chronic corticosterone treatment (30 day) of whole salamanders (Fig. 3). *LY-6E* was also upregulated by corticosterone in cultured tails, but is notably downregulated under chronic corticosterone treatment (Figs. 3 and 6).

We found that *A2M, FX,* and *TRX* were upregulated and *LY-6E* and *UMOD* were downregulated in regenerated tissues exposed to corticosterone for 14 days compared to controls (Fig. 5). This demonstrates that chronic exposure to corticosterone constitutively upregulates (*A2M*, *FX, TRX*) or downregulates (*LY-6E*, *UMOD*) genes across different stress durations and also during the process of regeneration.

### Transcriptional responses to temperature variability

**Fig. 7:**
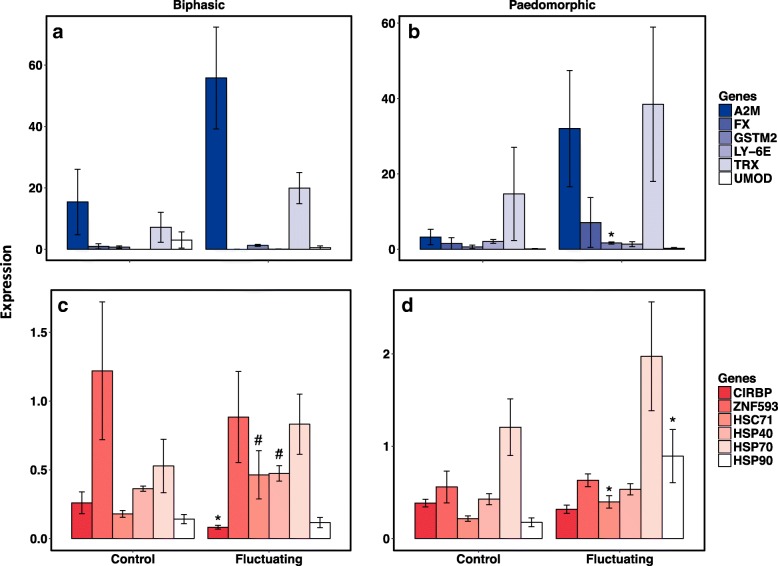
Relative qPCR expression values of six corticosterone sensitive genes (**a**,**b**) and six potential temperature sensitive genes (**c**,**d**) for adult biphasic (*N* = 12; a, c) and paedomorphic (*N* = 10; b, d) salamanders exposed to constant (21 °C) or fluctuating temperatures (μ = 21 °C, range 14–28 °C) for 4 weeks. Significant differences (*P* < 0.05) between temperature regimes are denoted with (*). Differences less than *P* < 0.01 are denoted with (#)

Adult paedomorphic and biphasic (metamorphosed) salamanders exposed to a thermally “stressful” environment with a 14 °C daily temperature fluctuation (21 ± 7 °C) for 4 weeks showed significant differential regulation of several genes compared to salamanders maintained at a constant temperature with the same mean (21 ± 0 °C). *HSC71* was significantly upregulated under fluctuating temperature regimes in the adults of both paedomorphic and biphasic salamanders. HSPs showed life cycle specific responses to fluctuating temperatures, with *HSP40* and *HSP70* upregulated in metamorphs, and *HSP90* upregulated in paedomorphs. Also, *CIRBP* was downregulated by temperature fluctuation in biphasic adults compared to those at stable temperatures (Fig. 7). With the exception of *GSTM2*, genes that were differentially regulated by chronic corticosterone (Fig. 3) did not differ in response to fluctuating temperatures (Fig. 7). In summary, some genes (e.g. *GSTM2*) are constitutively regulated by chronic corticosterone exposure and by fluctuating temperature. However, we identified almost a dozen genes with responses specific to corticosterone (*A2M*, *FX*, *LY-6E*, *TRX*, and *UMOD*) or fluctuations in temperature (*CIRBP*, *HSC71*, *HSP40*, *HSP70*, *HSP90,* and *ZNF593*).

## Discussion

The responses of species to changing climate are difficult to predict, and the environmental variables responsible for stress can be elusive. Compared to humans, there are relatively few metrics to assess the health of wildlife [4]. For amphibians this has been largely limited to body-weight metrics [42, 43], corticosterone levels [27–29, 35, 44, 45], and pathogen presence [46, 47]. Therefore it is important to develop more diverse metrics for understanding amphibian stress response and population health. Transcriptional responses of amphibians to stress hormones such as corticosterone have mostly focused on acute stress in the laboratory model *Xenopus* [36–38, 48, 49]. Studies that have investigated gene expression patterns associated with stressors that may not necessarily engage the HPI-axis have been limited to temperature [10, 40] and disease resistance [18, 50]. Here we show that chronic corticosterone exposure and temperature stress instigate unique transcriptomic patterns in the tail tips of a stream-dwelling salamander. Our study demonstrates an example of how transcriptomic data can provide useful information for understanding amphibian responses to diverse stressors in wild or captive populations.

Chronic activation of the HPI-axis and the subsequent production of glucocorticoids can have deleterious effects on immune function and increased vulnerability to disease [51–54]. We found that chronic corticosterone treatment altered the expression patterns of several genes involved in immune system response, oxidative stress, and tissue repair. In regard to the immune system, the upregulation of *LY-6E* is thought to be part of a compensatory mechanism against pathological dysfunction following infection [55, 56]. We found that *LY-6E* was strongly downregulated after chronic corticosterone treatment (Figs. 3 and 5). In comparison, *GSTM2* and *TRX,* which are known to combat cytotoxicity and oxidative stress [57, 58], were upregulated in our chronic corticosterone treatments of adult salamanders (Fig. 3). We also found that chronic corticosterone treatment upregulated *FX* and *A2M*, both of which are known to be involved in tissue repair [59–61]. Finally, *UMOD* (encoding Tamm-Horsfall glycoprotein) is best known for its diverse roles in the health of the mammalian kidney including immune functions and osmoregulation [62]. However, this gene is also expressed in diverse amphibian tissues [63], but the functional role of *UMOD* like proteins in amphibians is still uncertain. Thus far, *UMOD* has been shown to be upregulated in tadpole facial tissues in the presence of predators [64], downregulated in the tails of metamorphosing tadpoles (gene 18 in [65]), and downregulated in the skin of larval salamanders treated with thyroxin [66]. The expression of *UMOD* is strongly downregulated in response to corticosterone in the tail tips of adult and larval *E. tynerensis* in our study (Figs. 3 and 5).

Microarray analyses of *Xenopus* tails exposed to short-term (18 h) corticosterone showed differential regulation of 1968 genes [38]. We reduced their dataset down to 501 known genes and compared it to a subset of 265 genes from our corticosterone versus control analysis based on uncorrected *P*-values (α < 0.05). Nine genes were shared between their acute and our chronic corticosterone treatments, but six of these genes displayed opposite patterns of expression (Additional file 2: Table S2). Opposing patterns of gene expression may result from species-specific, durational (“acute” vs. “chronic”), or context dependent responses. For example, we found that *LY-6E* was strongly upregulated in response to acute corticosterone treatment (Fig. 6), but downregulated after chronic treatment (Figs. 3 and 5). The transcriptional responses of amphibians to elevated glucocorticoids are still a nascent area of research. However, this will likely be a fruitful avenue for future investigation, and has the potential to provide a promising alternative for analyzing corticosterone-based stressors in amphibians.

As previously noted, not all stressors necessarily engage the HPI-axis [13], and therefore stress assays based on glucocorticoids alone could overlook other important factors that can impact the health of an organism. We found several corticosterone regulated genes lacked differences in expression when adult salamanders were faced with a thermal challenge (Fig. 7). Among the temperature-regulated genes, some HSPs have been previously identified as responsive to temperature stressors [40, 67] and infection [68]. In particular, *HSP40*, *HSP70*, and *HSP90* are well known for their roles in the cellular response to heat stress [68], and HSPs have been suggested as potential biomarkers of heat stress [14]. We found that these genes, as well as *HSC71*, were upregulated in response to fluctuating temperature. Furthermore, HSPs have a variety of functions including minimizing cellular damage and maintaining homeostasis in a thermally fluctuating environment [68, 69].

We found that exposure to the stress hormone corticosterone or a thermally stressful environment produced unique and often consistent transcriptional patterns in salamanders. This suggests that transcriptomic patterns can be useful for monitoring the impact of diverse stressors. However, organisms are often simultaneously subject to multiple stressors [70], which can have additive or synergistic negative effects on amphibian health and survival [71–74]. Several of the genes that were assessed showed interactions between corticosterone exposure and heat stress (Table 2). The influence of corticosterone on *GSTM2*, *LY-6E*, *UMOD*, *CIRBP*, *ZNF593*, and *HSP90* appears to be temperature dependent, whereas the expression of *A2M*, *FX*, *CIRBP*, and *HSP40* appears to be influenced only by corticosterone or temperature.

**Table 2:** Adjusted *P*-values of the main effects, corticosterone and temperature, and the interaction obtained from two-way analysis of variance of qPCR expression values for each of the 12 candidate genes. Significant differences are designated with (^a^)

Gene	Corticosterone	Temperature	Corticosterone x Temperature
*A2M*	0.0033^a^	0.0999^a^	0.4132^a^
*FX*	0.0061^a^	0.4133^a^	0.7359^a^
*GSTM2*	0.0076^a^	0.4192^a^	0.0068^a^
*LY-6E*	0.0001^a^	0.2738^a^	0.0004^a^
*TRX*	0.0004^a^	0.9031^a^	0.4614^a^
*UMOD*	0.0001^a^	0.4320^a^	0.0001^a^
*ZNF593*	0.1637^a^	0.0055^a^	0.0078^a^
*CIRBP*	0.1035^a^	0.0001^a^	0.0283^a^
*HSC71*	0.9975^a^	0.0001^a^	0.2531^a^
*HSP40*	0.3875^a^	0.0030^a^	0.9810^a^
*HSP70*	0.0021^a^	0.0001^a^	0.5350^a^
*HSP90*	0.2095^a^	0.0073^a^	0.0005^a^

Transcriptomes provide a promising yet under-evaluated source of information for understanding the health of amphibian populations and identifying specific stressors. We show that several genes express consistent patterns of corticosterone or temperature regulation across life stages, life cycle modes, and even during tissue regeneration. We are not suggesting that the genes that we found to be differentially expressed by temperature and corticosterone exposure in *E. tynerensis* will necessarily exhibit the same patterns in other species. The expression response of a given gene to stress may be species or clades specific, and may also show differences among sexes and tissues (e.g. [37, 75]). Furthermore, just like other biomarkers, there are a number of factors that may need to be resolved for each system before meaningful interpretation can be drawn about wild populations [4, 15]. Initial experiments would ideally be conducted under controlled settings and would entail exposing individuals to one or more stressors and biopsying an accessible tissue for transcriptomic analyses. This is to identify candidate genes that could be further evaluated under a range of contexts (stages, sexes, etc.). Ontogenetic assessment will be particularly important for amphibians that undergo dramatic metamorphosis between life stages, which involves significant changes to endogenous glucocorticoid levels [6, 34, 35] and gene expression patterns [37, 38]. If consistent stress response patterns ultimately emerge across clades, then qPCR of previously identified candidate genes for related species, instead of transcriptomics, could be used to reduce costs of development and assessment.

When facing a stressful environment, the immediate response of a healthy organism is often to compensate for or protect against the stressor [33]. This can involve behavioral, physiological, and molecular responses, which normally subside once the stressor is removed. The impact of the stressor(s) on the health of the organism can vary based on intensity and duration. One of the greatest challenges in deciphering the patterns of any biomarker is to know when a value represents a normal (healthy) response to an acute stressor versus when the system is compromised by severe and/or chronic stress [4]. Furthermore, organisms also exhibit geographic variation in baseline levels of stress hormones [76] as well as transcriptomes [77], and can evolve (adapt) to changing conditions [15]. Therefore, geographic variation and adaptation may not necessarily indicate chronic stress. These aspects need to be considered when developing assays for stress, especially across wide ranging species.

## Conclusions

Amphibians are negatively responding to ever increasing environmental stressors such as habitat loss, disease, and climate change. Using a transcriptomic approach, we identified a panel of genes that consistently and persistently responded to exposure of the “stress” hormone corticosterone and temperature variation across developmental stages, life cycle modes, and during tissue regeneration. While the specific genes identified here may be only relevant to this species, our study suggests that transcriptomics could be used to identify suites of genes that are indicative of the health of wild amphibian populations. Integrating transcriptomic analyses with other metrics of population stress and health expands the toolkit for conservation and management for understanding the factors that lead to amphibian declines.

## Methods

### Specimens

The Oklahoma Salamander (*Eurycea tynerensis*) inhabits small streams in the Ozark Plateau of east-central North America, and exhibits alternative life cycle modes. Most populations of *E. tynerensis* have aquatic larvae that metamorphose into terrestrial adults (biphasic), whereas others forego metamorphosis and maintain their aquatic larval morphology and ecology into adulthood (paedomorphosis) [78, 79].

Some of our experiments are based on wild-caught *E. tynerensis*, while other experiments are based on F_1_ generation larvae and adults raised under controlled conditions in the lab. Prior to each experiment, both wild-caught and captive raised salamanders were acclimated at 18 °C for at least 2 days. For all experiments, larval and paedomorphic salamanders were maintained individually in 500 ml of their assigned solution; biphasic salamanders were kept on wet paper towels. Each experiment was conducted in incubators and experimental solutions were replaced and salamanders were fed bloodworms (chironomid larvae) every other day. All food provided was consumed across all experiments. To avoid disturbance the specimens were intentionally isolated, but this meant that no behavioral data were collected.

At the conclusion of the transcriptome experiment, salamanders were euthanized in a 0.1% solution of tricane methanosulfate (MS-222). For all other experiments salamanders were anesthetized by immersion in a 0.05% solution of MS-222 and awoken with dechlorinated tap water. The tail-tip (< 10% of tail) was dissected off, snap-frozen on dry ice, and stored at − 80 °C. Salamander care, maintenance, and experimentation were approved by the University of Tulsa (IACUC protocol TU-0028), and all experiments were performed in accordance with this protocol.

### Experimental designs

Organisms experience a variety of stressors and may respond by expressing unique quantifiable symptoms. For example, temperature stress induces the expression of Heat Shock Proteins (HSPs) [14, 80] that may be independent of the HPI-axis. To evaluate whether genes show differential transcriptional responses to specific stressors, our first experiment was designed to test whether chronic corticosterone treatment and different temperature regimes would provide transcriptional signatures in a conveniently biopsied tissue (salamander tail tip [10]; Table 1). Wild caught adult male paedomorphic *E. tynerensis* (*N* = 24) collected from the same locality were randomly split between incubators set at either 11 or 21 °C. Paedomorphic populations of *E. tynerensis* are adapted to relatively cool streams, and the population used in this experiment is from a groundwater fed stream with an average temperature of 13 °C (range 8 °C to 19 °C; Treglia et al. *in prep*). These salamanders tend to move to cooler microhabitats, deeper into the streambed when summer temperatures reach upper limits. Over the course of the month long experiment, salamanders kept at 11 °C maintained their body weight, while the body weights of those kept at 21 °C were reduced by ~ 16%. Therefore, 21 °C is above their normal temperature range and was considered a thermal stressor, while 11 °C was not considered stressful.

Within each temperature regime, half were exogenously treated with 100 nM corticosterone for 30 days. This dose is within the upper range or slightly above plasma corticosterone concentrations measured in other salamanders [44, 45]. Ethanol was used as a vehicle for corticosterone, so an equivalent amount of ethanol (< 0.001%) was added to control water. Due to the small size of the animals (average 370 mg), we were unable to obtain sufficient blood plasma to estimate circulating corticosterone levels at the conclusion of the experiment. However, larva and paedomorphic amphibians breathe through their porous skin and gills, and amphibians bathed in exogenous corticosterone solution readily up-take this hormone into their system (reviewed in [26, 81]). We replaced the corticosterone solution every other day across the course of the experiment to ensure a continuous dose.

Variation in physiological processes can be highly dependent upon age; therefore, gene expression patterns of adults may be different from larvae and juveniles [8, 35]. Furthermore, amphibians often exhibit variation in life cycle patterns [82], including discrete polymorphisms as observed in *E. tynerensis* [78, 79]. We conducted a series of experiments to validate the efficacy of corticosterone-regulated genes under different stages and life cycle modes using lab-raised larvae from paedomorphic and biphasic populations. Finally, wild amphibians commonly lose and regenerate their tails. Therefore, ideal biomarkers should produce consistent patterns even when tissues have been regenerated. We tested whether corticosterone induced transcriptional patterns were reproducible in newly regenerated tissues by analyzing tail clips that were regenerated while chronically exposed to corticosterone for 2 weeks.

During these experiments larvae from paedomorphic (*N* = 12) and biphasic (*N* = 18) populations were borne and raised in the laboratory at 21 °C. These larvae were exposed to either 100 nM of constant corticosterone or control (filtered water) for 28 days. After the first 14 days, 6 larvae from biphasic populations were anesthetized by immersion in MS-222 and their tail tips were biopsied for gene expression, and the salamanders were returned to 100 nM corticosterone treatment. This provided an earlier time point of corticosterone exposure (2 weeks), and also to evaluate the effects of corticosterone on expression patterns during regeneration. After 14 more days (at 28 days from the start of the experiment) tail tips were removed from all salamanders including the regenerated portion of the tails that were previously biopsied at 14 days.

We also conducted a tissue culture experiment on excised tail tips from 12 lab-raised adult, but non-reproductive (18 month old) paedomorphic *E. tynerensis* to test the effects of corticosterone on transcription when tissues are isolated from the rest of the endocrine system. The distal portions of tails (< 25% of total lengths) were cultured at 21 °C in 6-well plates and bathed in Leibovitz L-15 solution (diluted 2:1) with penicillin/streptomycin (100 units per ml). Tails were treated with either 100 nM corticosterone diluted in EtOH or an equivalent amount of EtOH as a control. Treatment solutions were replaced every 24 h. After 48 h of exposure to treatment conditions, tail tips were rinsed with 1x PBS and snap frozen on dry ice and stored at − 80 °C until RNA extraction.

We used paedomorphic (*N* = 9) and biphasic (*N* = 8) adult wild-caught salamanders to test for gene expression differences when exposed to a “stressful” thermal regime that involved dramatic daily changes in temperature. Salamanders were randomly split between 30 day temperature treatments, constant 21 °C or a thermally stressful fluctuating temperature regime with an average of 21 °C and a cyclical daily range of 14–28 °C.

### RNA extraction, transcriptome sequencing, and quantitative PCR

RNA was isolated from tail tip tissue using Trizol Reagent (Invitrogen, Carlsbad, CA) following the manufacturer’s protocol. RNA concentrations were determined using either a QuBit fluorometer 2.0 (Thermofisher Scientific) for RNA-Seq samples, or a NanoDrop 8000 for samples that would be analyzed by quantitative PCR (qPCR). RNA-Seq libraries were prepared using the TruSeq RNA Library Preparation Kit (Illumina) and sequenced using 300 or 500 cycle V2 paired end read kits on an Illumina MiSeq at the University of Tulsa. All reads with a Q score less than 30 were discarded and adaptors were trimmed using MiSeq Reporter prior to analyses.

We have been iteratively building a partial *Eurycea tynerensis* transcriptome based on diverse tissues from several larval and adult individuals. These tissues included adult tail tips (*N* = 24), adult skin (*N* = 30), larval brains (*N* = 2), adult brains (*N* = 2), larval livers (*N* = 2), adult kidneys (*N* = 1), oviducts (*N* = 1), and testes (*N* = 1). We performed de novo assemblies of each tissue type and of individuals using CLC Genomics Workbench version 7.5.1 (Qiagen). These assemblies included a total of more than 100 million 150 to 250 bp paired-end Illumina reads, and were assembled with a similarity fraction of 0.95 or higher. Consensus sequences were extracted using a minimum coverage of 5x and ambiguity threshold of 0.25. We primarily identified genes by individually BLASTx searching transcripts against NCBI’s non-redundant protein database (parameters: organism = Vertebrata or Amphibian; max target sequence = 100; expected threshold = 10; max word size = 6; matrix = BLOSUM62; filter = low complexity regions). Groups of similarly identified transcripts were aligned using Clustal Omega and their uniqueness was evaluated by visually inspecting alignments. We identified 4348 transcripts with unique coding sequences (presumably non-redundant genes) totaling ~ 10.9 million bp, which was used as the reference for transcriptomic analyses.

We used the RNA-Seq function in CLC Genomic Workbench (95% similarity, 50% length fraction) to map 18.1 million, 150 bp paired-end, pass-filtered reads (~ 754 K reads per sample) to the partial *E. tynerensis* transcriptome (4348 genes). Our number of reference transcripts and depth of sequencing was sufficient to identify a large number of “highly expressed” genes that show large disparities among stress treatments. This made them readily quantifiable via qPCR. We used *EdgeR* [83] in the statistical platform R version 3.4.0 [84] to identify differentially expressed genes between treatment groups (corticosterone or temperature) based on total read counts. To determine differentially expressed genes, we first reduced our initial 4348 genes to 4082 based on a minimum of 1 count per million across at least 6 of our 24 RNA-Seq libraries. The ‘calcNormFactors’ function was used to normalize each sample library based on scaling factors that minimize the log-fold changes between each sample. We used the ‘estimateDisp’ function to fit negative binomial models based on weighted likelihood empirical Bayes method to determine dispersion estimates for each sample. The ‘decideTests’ function was used to assess differential expression with a Benjamini-Hochberg adjusted *P*-value of 0.05 to minimize false discovery rates. A heat map was plotted using ‘hclust’ [84] and ‘heatmap.2’ in R [85] to assess the degree of clustering among treatment groups.

TaqMan BHQ1a-6FAM qPCR assays were developed for 12 differently regulated genes (Additional file 1: Table S1; see Results section). This included six corticosterone regulated genes: Alpha-2 Macroglobulin (*A2M*), Coagulation Factor X-like (*FX*), Glutathione-S Transferase Mu 2 (*GSTM2*), Lymphocyte Antigen 6E (*LY-6E*), Thioredoxin (*THIO*), Uromodulin-like (*UMOD*), and six temperature regulated genes: Cold Inducible RNA Binding Protein (*CIRBP*), Zinc Finger 593 (*ZNF593*), Heat Shock Cognate 71 (*HSC71*), and the Heat Shock Proteins 40, 70, and 90 (*HSP40*, *HSP70*, and *HSP90*). cDNA was synthesized using SuperScript II (Invitrogen) and random hexamer. Reactions for qPCR were run with ABI TaqMan Gene Expression Master Mix on an ABI StepOne Plus (Thermofisher Scientific). Samples for a given gene were run simultaneously with a five-point standard curve, negative RT reactions, and negative controls. Expression quantity values were interpolated from CT values (number of cycles) based on the standard curves for each gene. Expression values were normalized with ribosomal protein L8 (*rpL8*), which is commonly used for normalization in amphibian gene expression studies [86, 87]. Relative gene expression values were log transformed and significant differences among groups were determined using ANOVA and a multiple-tests adjusted *P*-value (Benjamini and Hochberg method) in the R statistical platform [84].

## Additional files

Additional file 1: **Table S1.** Primers and probes for quantitative RT-PCR. (DOCX 18 kb)

Additional file 2: Results of differential expression analyses. (XLSX 1844 kb)

## Data Availability

Public access to databases is open. RNA-Seq reads are available on Genbank (BioProject PRJNA531501), and log fold changes for each experiment and each gene are available as supplemental files.

## Abbreviations

ANOVA: Analysis of variance
bp: base pairs
cDNA: Complimentary deoxyribonucleic acid
HPI-axis: Hypothalamic pituitary interrenal-axis
HSP: Heat shock protein
MS-222: Tricane methanosulfate
nM: nanomolar
qPCR: quantitative Polymerase Chain Reaction
RNA-Seq: Ribonucleic acid sequencing
