# Supplementary material for: Multiple stressors produce differential transcriptomic patterns in a stream-dwelling salamander
Source: BMC Genomics. 2019 Jun 11;20:482. doi: 10.1186/s12864-019-5814-y (PMC6560913; doi:10.1186/s12864-019-5814-y)
Supplement: Supplementary file 1 — Table S1. Primers and probes for quantitative RT-PCR. (DOCX 18 kb) [file 12864_2019_5814_MOESM1_ESM.docx]

Additional file 1: Table S1 Primers and probes for quantitative RT-PCR

| **Gene** | **Primers** | **TaqMan probe** |
| --- | --- | --- |
| *A2M* | 5’-GTTGTGGCGAACAGATCCTAT-3’ | 5’-ATTACTTGATGTCCGTGGGCAGCT-3’ |
|  | 5’-CTGGCATATCCGCCATTGA-3’ |  |
| *FX* | 5’-GATGTGGTGTCTGGATTTCCTA-3’ | 5’-CCTTGCAGGTTGAACAAATCGCACC-3’ |
|  | 5’-CACGTTCATTATCCAGTTGCG-3’ |  |
| *GST* | 5’-GCTGGAGTACACCGGAGA-3’ | 5’-CGCTAGGGCTGGACTTCCCAAA-3’ |
|  | 5’-GCCGTCAATCAGATATGGAAGT-3’ |  |
| *LY-6E* | 5’-GACCTGGAAGTCTTCTGGAATC-3’ | 5’-CAAATGGGCACGCGTTTAGAGTCC-3’ |
|  | 5’-GGGCATCATTGGCCTACTAC-3’ |  |
| *TRX* | 5’-GCCTCCTCTGTGTTCACTTT-3’ | 5’-ACATCTGGGCGCTCGTCACATAAA-3’ |
|  | 5’-GCTTGTACTGGTGTACTACTCTTC-3’ |  |
| *UMOD* | 5’-GCGCTGGACATGGTAATTAGT-3’ | 5’-TCCAGTACCTCCAACATTCAATGTCAAC-3’ |
|  | 5’-GCGGTATGAGGCCATAGTTAAT-3’ |  |
| *CIRBP* | 5’-GCCAAGTTTGGGAACATATCAG-3’ | 5’-AAAGACCGGGAGACCCAGAGATCC-3’ |
|  | 5’-TCAAACGTGACAAAGCCAAAG-3’ |  |
| *ZNF593* | 5’-TGAACCAGGAGGTGGACTT-3’ | 5’-AACGCCCAGCACTACTGCCT-3’ |
|  | 5’-TCGCAACTGCTTTAGCCTT-3’ |  |
| *HSC71* | 5’-TTGTACTGGTCAGCCTCCT-3’ | 5’-CGGCCTTTGTCATTGGTGATGGTG-3’ |
|  | 5’-GCAGTGGATAAGAGTACTGGAAAG-3’ |  |
| *HSP40* | 5’-GGACAGTAGAACTATCGTCATAACA-3’ | 5’-TGGACAAATAGTCAAGCACGGTGATGT-3’ |
|  | 5’-CTGCGATAGACGGGCATAC-3’ |  |
| *HSP70* | 5’-AGCAGATTGTTGTCCTTGGT-3’ | 5’-CCGCTCACCTTCGTACACCTGAAT-3’ |
|  | 5’-GCAGACCTTCACCACATACTC-3’ |  |
| *HSP90* | 5’-GAGTATGGCGAGTTCTACAAGAG-3’ | 5’-ACCAATGACTGGGAGGACCACTTG-3’ |
|  | 5’-GCGTGGGATGAACAGCA-3’ |  |
